# Hemolytic anemia following intravenous immunoglobulins in children with PIMS-TS: Two case reports

**DOI:** 10.3389/fped.2023.1144914

**Published:** 2023-04-11

**Authors:** E. Sedlin, S. Lau, H. von Bernuth, T. Kallinich, B. Mayer

**Affiliations:** ^1^ Pediatric Respiratory Medicine, Immunology and Critical Care Medicine, Charité-Universitätsmedizin Berlin, Berlin, Germany; ^2^ Klinik für Kinder-und Jugendmedizin, Helios Klinikum, Berlin-Buch, Germany; ^3^ Comprehensive Allergy Center Charité (CACC), Charité-Universitätsmedizin Berlin, Berlin, Germany; ^4^ Department of Immunology, Labor Berlin GmbH, Berlin, Germany; ^5^ Berlin Institute of Health (BIH) at Charité-Universitätsmedizin Berlin, Berlin, Germany; ^6^ BIH Center for Regenerative Therapies (BCRT), Berlin Institute of Health (BIH), Berlin, Germany; ^7^ Deutsches Rheuma-Forschungszentrum (DRFZ), An Institute of the Leibniz Association, Berlin, Germany; ^8^ Institute for Transfusion Medicine, Charité-Universitätsmedizin Berlin, Berlin, Germany

**Keywords:** SARS-CoV-2, PIMS-TS, MIS-C, hemolytic anemia, blood group, IVIG (intravenous immunoglobulin) administration

## Abstract

This is the first case report on two children presenting with immediate and severe hemolytic anemia following the administration of high-dose intravenous immunoglobulins (IVIGs) in the context of pediatric inflammatory multisystem syndrome temporally associated with SARS-CoV-2 (PIMS-TS). Hemolytic anemia was described as a significant decrease in hemoglobin and an increase in lactate dehydrogenase after the second administration of high-dose IVIGs was performed. Both patients were found to have AB blood group. One of our patients showed massive pallor, weakness, and inability to walk in association with hemolysis. However, in both cases, the anemia was self-limiting and transfusion of red blood cells was not required: both patients recovered without persistent impact. Nonetheless, we aim to draw attention to this widely unknown adverse effect of IVIG, especially in the context of PIMS-TS. We suggest determining the patient’s blood group prior to high-dose IVIG infusion and replacing the second IVIG through high-dose steroids or anticytokine therapy. Using IVIGs containing lower titers of specifically anti-A or anti-B antibodies to avoid isoagglutinin-caused hemolytic anemia is desirable; however, the information is not routinely available.

## Introduction

Pediatric inflammatory multisystem syndrome temporally associated with SARS-CoV-2 (PIMS-TS) is a severe hyperinflammatory condition with multiorgan involvement, also known as multisystem inflammatory syndrome associated with coronavirus disease 2019 (MIS-C), developing about 3–6 weeks after an acute or subclinical SARS-CoV-2 infection. Data on the incidence of PIMS-TS are rare, but PIMS-TS is reported to occur in approximately 4.5 per 10,000 children infected with SARS-CoV-2 in Great Britain (1).

PIMS-TS presents with symptoms resembling Kawasaki disease (KD) (2). PIMS-TS is not contagious and represents a delayed hyperinflammation process with cytokine storm (3). Levels of C-reactive protein (CRP), ferritin, and D-dimers, for example, have been reported to be higher in PIMS-TS than those in KD and represent the overactivation of the immune system (3, 4). The diagnosis of PIMS-TS can be confirmed if the following criteria are met: (1) fever > 48 h, (2) elevated inflammatory values such as C-reactive protein, (3) at least two-organ involvement (conjunctivitis, exanthema, gastrointestinal symptoms like diarrhea, abdominal pain, and others), (4) positive SARS-CoV-2 polymerase chain reaction (PCR) or point of care (PoC) antigen tests, positive SARS-CoV-2 antibodies (IgM or IgG), or recent contact with an infected person with SARS-CoV-2, and (5) exclusion of other causes of infection (5). Hyperinflammatory conditions like KD and PIMS-TS are often treated with immunomodulating therapeutic measures like IVIGs. Side effects are mainly headache and hyperviscosity. Hemolytic anemia has been reported in association with high-dose treatment with IVIG; however, high concentrations of isoagglutinins were identified as causal factors in patients with Kawasaki disease and non-O blood groups (6). Others reported autoimmune responses to blood group antigens (7). This is the first case report on two children presenting with hemolytic anemia following the administration of IVIGs in the context of PIMS-TS.

## Case presentation

Patient 1, a 2-year-old girl, presented to our emergency department with a high fever of up to 40°C for 5 days, abdominal pain, irritability, and glazed lips. Immediately before admission, she also had a sore throat, mildly enlarged submandibular lymph nodes, and a red, target-shaped efflorescence on the right cheek. Her height, weight, and body mass index were at the 89th, 86th, and 69th percentiles, respectively. The child had an elevated pulse rate during fever episodes and age-appropriate blood pressure. Clinical examination revealed regular auscultation of the heart and the lung and no signs of hepatosplenomegaly, which was confirmed by abdominal ultrasound. The initial CRP was elevated, with levels up to 140 mg/ml, and D-dimers surpassed the reference range >35 mg/L (Table 1). Furthermore, lymphopenia (19%, normal range 22–59) and neutrophilia (75%, normal range 25–74) were noticed. A known SARS-CoV-2 contact was reported 3 weeks before; however, only minor symptoms were observed afterward. The further workup with echocardiography showed a conspicuous but minimal dilatation of the left coronary artery (3 mm). The ejection fraction was normal. The heart ultrasound normalized 9 months after admission. The patient did not have any pre-existing conditions. Antipyretics but no other medications were administered prior to hospital admission. The psychosocial circumstances were unremarkable, with normal development.

**Table 1:** Characteristics.

	Patient 1: 2 years, 4m-old female			Patient 2: 8 years, 4m-old female		
Onset coronavirus infection	03/2021			08/2021		
Onset PIMS-TS	∼3 weeks after infection			∼3 weeks after infection		
Clinical appearance of PIMS-TS	Recurrent fever episodes > 40°C without response to antipyresis for 5 days, sore throat, submandibular lymph nodes, abdominal colics; irritability; glazed lips, target-shaped efflorescence on right cheek			Recurrent fever episodes up to 39°C for 3 days, dry cough, headache, neck pain, sore throat, cervical lymphadenopathy, swollen tonsils, suspected meningism, chest pain		
Blood group	AB, RhD-positive			AB, RhD-positive		
Irregular isoagglutinins	Anti-A1/anti-A2			Anti-A1		
DAT	IgG+			IgG+		
	Laboratory at admission	Laboratory 5 days after 2nd IVIG	Laboratory 30 days after 2nd IVIG	Laboratory at admission	Laboratory 3 days after 2nd IVIG	Laboratory 20 days after 2nd IVIG
SARS-CoV-2 IgG/IgA ELISA	Positive/positive	—	—	Positive/positive	—	—
Hemoglobin (11.2–14.6 g/dl)	10.4^a^	5.7^a^	12.6	11.5	6.8^a^	8.9^a^
LDH (120–300 U/L)	247	1101^a^	397^a^	239	486^a^	307^a^
Haptoglobin (0.3–2 g/L)	—	<0.1^a^	—	—	<0.1^a^	<0.1^a^
CRP (<5 mg/ml)	140^a^	4.9	<1	243^a^	55.9^a^	1.7
Ferritin (7–84 µg/L)	470.3^a^	—	43.8	257.3^a^	730.7^a^	221.7^a^
D-dimers (<0.50 mg/L)	>35^a^	0.51^a^	>35^a^	1.12^a^	2.99^a^	—
Factor VIII (70%–150%)	204^a^	—	—	275^a^	—	—
vW antigen (56%–162%)	226.6^a^	—	—	291.9^a^	—	—
Troponin T (<14 ng/L)	<3	<3	—	22^a^	7	—
NT-pro-BNP^b^ ng/L—*no references*	139	399	—	15,960	1,656	—

PIMS-TS, pediatric inflammatory multisystem syndrome temporally associated with SarsCov2; DAT, direct antiglobulin test; IVIG, intravenous immunoglobulin; CRP, C-reactive protein; LDH, lactate dehydrogenase.

^a^ Out of reference range.

^b^ Age-dependent reference range for NT-pro-BNP.

Patient 2, an 8-year-old girl, was admitted to our emergency department with a fever of up to 39°C for 3 days, dry cough, chest pain, headache, and neck pain with cervical lymphadenopathy and suspected meningism as well as tonsil swelling. The strep A test was negative in the emergency room. Her height, weight, and body mass index were at the 91th, 80th, and 63th percentiles, respectively. An elevated pulse rate during fever episodes and subnormal blood pressure were noticed. The clinical examination revealed regular lung and heart auscultation and a slightly distended abdomen. The abdominal ultrasound did not reveal hepatosplenomegaly. In addition to discrete palmar erythema, no skin lesions were noticed. The patient suffered from a reduced general condition. CRP levels were elevated up to 243 mg/ml. Blood cell count showed up to 84% neutrophilia and lymphopenia with the lowest value of 15%. Cerebrospinal fluid results were unremarkable. Vitamin D3 level was low (14 nmol/L). A known SARS-CoV-2 contact was reported approximately 3 weeks before. Echocardiography was initially normal, with the ejection fraction at the lower limit of normal. However, pro-BNP was elevated (15,960 ng/L). Soon, a mild dilatation of the coronary arteries (3–4 mm) and a minor pericardial effusion were observed. The ejection fraction was normal. The mean arterial pressure (MAP) was decreased by 46 mmHg. The heart ultrasound normalized already 7 days after admission. Patient 2 did not have any pre-existing conditions or regular medications. She lived with a single mother and, as far as known, was of unremarkable psychosocial condition and normal development.

We diagnosed PIMS-TS according to patients’ symptoms and laboratory parameters, such as elevated CRP, neutrophilia and lymphopenia, markers of acute-phase reactions (factor VIII, vW antigen), D-dimers, and especially positive SARS-CoV-2 IgG antibodies, presumably due to reported SARS-CoV-2 some weeks before (Table 1). Both patients showed two affected organ systems. Furthermore, we excluded other causes of infections like potential bacterial and virus infections, e.g., cytomegalovirus, Epstein–Barr virus, and adenovirus.

**Figure 1:**
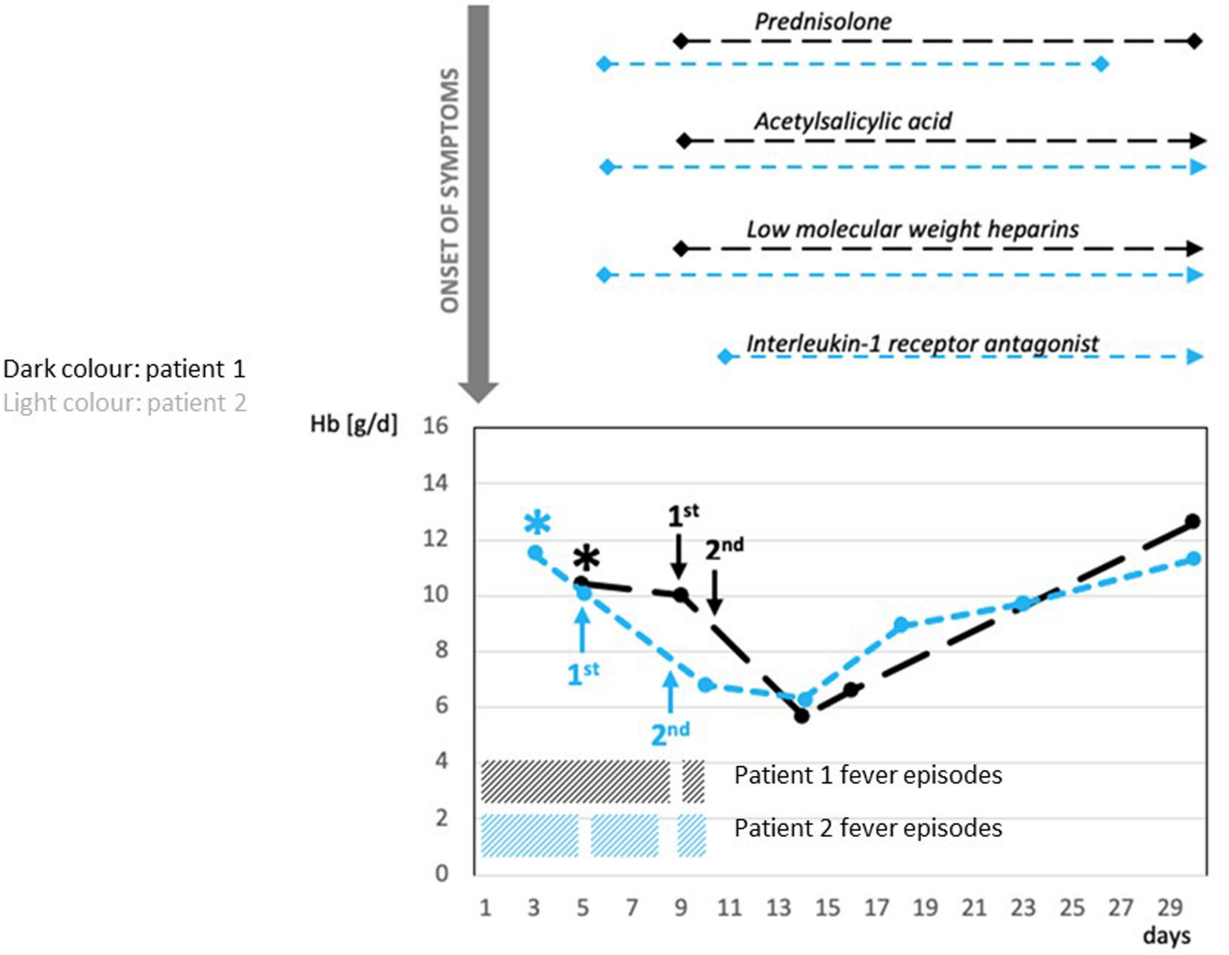
Correlation between administration of IVIG and severe hemolytic anemia: time course of Hb, treatment of PIMS-TS, and recurrent fever episodes. *admission to hospital; *Prednisolone* (2 mg/kg/day): Patient 1 and Patient 2 for 3 weeks; *Acetylsalicylic acid* (5 mg/kg b.w./day): Patient 1 for 10 months, Patient 2 for 5 weeks; *Low-molecular-weight heparins* (1 mg/kg b.w./d): Patient 1 for 3 weeks, Patient 2 for 5 weeks; *Interleukin-1 receptor antagonist* (anakinra—100 mg subcutaneously/3 times/week): Patient 2 for 4 weeks. Patient 1: first administration of IVIG 2 g/kg—9 days after onset of PIMS-TS; second administration of IVIG 2 g/kg about 40 h after the first administration. Patient 2: first administration of IVIG 2 g/kg 5 days after onset of PIMS-TS; second administration of IVIG 2 g/kg 4 days after the first administration. PIMS-TS, pediatric inflammatory multisystem syndrome temporally associated with SARS-CoV2; IVIG, intravenous immunoglobulin; Hb, hemoglobin.

Initially, both patients received intravenous broad-spectrum antibiotic therapy due to a high fever of unknown origin. Additionally, patient 2 received antiviral medications due to suspected encephalitis at admission. Following confirmation of PIMS-TS, anti-inflammatory treatment with prednisolone (2 mg/kg b.w./day) was administered, in addition to acetylsalicylic acid (5 mg/kg b.w./day), due to the mildly enlarged coronary arteries and prophylactic subcutaneous low-molecular-weight heparins (1 mg/kg b.w./day). The initial dose of IVIGs (2 g/kg b.w./day) was administered within the first 4 days after admission (Figure 1).

Anti-inflammatory treatment with prednisolone was given for almost 3 weeks to both patients. Cardioprotective acetylsalicylic acid was administered for 10 months to patient 1 and for 5 weeks to patient 2. Subcutaneous low-molecular-weight heparins were administered for 3 weeks to patient 1 and for 5 weeks to patient 2.

After a short, afebrile period, both patients suffered a relapse with high fever and reduced general condition. Therefore, both received a second administration of IVIGs within 10 days after the onset of symptoms (Figure 1). Patient 1 received the second IVIGs approximately 40 h after the first administration, whereas patient 2 received the second IVIG administration 4 days after the first dose (Figure 1).

Subsequently, patient 1 presented with symptomatic hemolytic anemia with pallor, massive weakness, inability to walk, and decreased hemoglobin (Hb) (from 10.4 to 5.8 g/dl), increased lactate dehydrogenase (LDH) (247–1,101 U/L), and nondetectable haptoglobin (Table 1, Figure 1). Patient 2 developed a complicated course, requiring intermediate care (IMC) for 6 days due to blood pressure instability. Being immobilized on IMC, the symptoms of hemolytic anemia were clinically not as evident in patient 2 as in patient 1. Beyond that, patient 2 suffered from edema, requiring the intake of diuretics. Furthermore, patient 2 received an interleukin-1 receptor antagonist (anakinra; 100 mg subcutaneously/three times/week) for 4 weeks due to a further recurrent fever episode after the second administration of IVIGs. In both patients, the anemia was self-limiting and did not require transfusion of red blood cells (RBCs). At the last follow-up (10 months after the onset for patient 1, 5 months after the onset for patient 2), both patients were symptom-free and did not take any medication.

The blood group of both children was determined as AB RhD-positive. However, irregular isoagglutinins were detectable in the plasma of both children (anti-A1 and anti-A2 in patient 1 and anti-A1 in patient 2). In addition, the direct antiglobulin test (DAT) was positive for IgG in both patients, leading to the diagnosis of IVIG-associated hemolysis. No other irregular allo- or autoantibodies to RBCs were detected in the plasma or eluate of either patient. The anti-A isoagglutinins were most likely transmitted with the IVIG batch, causing severe hemolysis. Subsequently, we compared the anti-A and anti-B titers of the administered preparation with another commercially available preparation. The anti-A1 IgG titers of 1:128 were significantly higher in the preparation that both patients received than the anti-A1 titers (1 : 16) in an IVIG preparation from another manufacturer.

The outcome of both children during the follow-up of 10 and 5 months, respectively, was excellent. Patient 1 could be discharged home after almost 2 weeks of inpatient treatment, and patient 2 could be discharged home after almost 3 weeks. Thus, the hemolysis due to the treatment with IVIGs did not prolong the hospital stay. The patients and parents were fully informed about hemolysis as a potential side effect of IVIGs.

Both children were seen for regular follow-ups including blood tests and echocardiography in our outpatient clinic after discharge. There were no long-term consequences regarding anemia, inflammatory values, physical conditions, or pathologies in the heart ultrasound. However, patient 2 suffered from fatigue for another 6 weeks after discharge.

## Discussion

IVIG-associated hemolysis appears to be associated with high-dose IVIG >1 g/kg, non-O blood group, and severe inflammatory disease (6, 7). We emphasize that blood group AB, as in both our patients, shows the highest risk of developing hemolytic anemia and that blood group O is almost unaffected in IVIG-associated hemolysis (6). The prevalence of IVIG-associated hemolysis emerged with newer IVIG preparations after 2007, and the prevalence of IVIG-associated hemolysis, especially in KD, shows a variation between 0.36% and 16% (6). Pathophysiologically, IVIG products presumably cause opsonization of erythrocytes, undergoing FcgRIIa-dependent phagocytosis by activated macrophages, leading to extravascular hemolytic anemia. This opsonization *via* isoagglutinins anti-A and anti-B subclass IgG2 in IVIG products is enhanced in the presence of proinflammatory cytokines (6). Thus, inflammatory disorders like PIMS-TS and KD enhance the potential risk of isoagglutinin-mediated hemolysis. Furthermore, high anti-A-IgG titers in IVIGs can provoke hemolysis (8). Although patient 2 showed a slight decrease in hemoglobin before the start of IVIGs, assumingly due to the severe inflammation, hemolysis played the major role in the decrease of hemoglobin.

In KD, in addition to alloimmune hemolysis by passively transferred isoagglutinins (anti-A and anti-B), a potentially autoimmune-mediated mechanism by anti-M or anti-C/-c antibodies has been postulated in individual case reports (7, 9). However, in these publications, hemolysis lasted much longer than in our two cases, and blood transfusion was needed in one case, indicating a more severe clinical presentation. Furthermore, in our two children, additional irregular allo- or autoantibodies to blood groups were excluded.

Referring to the data from the Italian observational multicenter retrospective study comparing KD and KD-like multi-inflammatory syndrome diagnosis in association with SARS-CoV-2 (KawaCOVID), our patients fitted into the KawaCOVID group based on the presence of persistent fever (>48 h), lymphopenia, and evidence of single- or multiorgan dysfunction (2). Neither child showed major skin symptoms; the younger child (case 1) had only mild cheilitis and a single target-shaped efflorescence on the right cheek. The older child had very discrete palmary erythema. Patient 2 showed hypotension, which was rarely seen in KD, and required intermediate care.

The superiority of an immediate prednisolone therapy for PIMS-TS, as performed in the reported children, in addition to the initial IVIG application, has been demonstrated (10, 11). At the time our two patients were admitted to the hospital, the treatment guidelines for Kawasaki-like PIMS-TS recommended the use of high-dose methylprednisolone, an anticytokine therapy (e.g., anakinra, tocilizumab, or infliximab), or a second IVIG dose in the case of an insufficient response to the first IVIG application (12, 13). Based on the observed significant adverse effects of the second IVIG application, published data, and our own experience, we would currently recommend the use of high-dose steroid therapy or anakinra instead of the second IVIG dosage (14, 15). If the second dose of IVIGs is still indicated, we advocate intervals of ≥ 48 h between the first and second doses to reduce the cumulative dose per time and to have the chance of early detection of potential hemolysis after the first dose.

In doing so, we refer especially to possible high-risk patients with AB blood group. We propose ABO blood group typing *before* the administration of high-dose IVIGs to first carefully evaluate the indication of IVIG administration in AB blood group and second in the case of subsequent blood transfusion because of potential isoagglutinins counteracting blood group analysis. The current requirements for the maximum titer (1:64) of anti-A and anti-B in IVIG preparations should also be considered by the manufacturers and authorities (6). Furthermore, we suggest monitoring patients with severe inflammatory syndromes carefully for hemolytic anemia 3–5 days after IVIG administration regarding Hb, LDH, and haptoglobin.

If hemolysis occurs after high-dose IVIGs and autoantibodies against RBCs are detected in addition to a positive DAT, autoimmune-mediated hemolysis should also be considered a rare differential diagnosis (7, 9), especially if it is long-lasting and severe.

We conclude that IVIG-associated hemolysis can appear more probably after the second administration of IVIGs within less than 48 h (dose per time) due to a high cumulative effect. Additionally, we assume that IVIG-associated hemolysis could be caused by a single high-dose IVIG if there are excessive isoagglutinins in the batch (>1:64), especially anti-A.

Our case reports should be considered in light of several limitations. First, we emphasize that our observation of hemolytic anemia following IVIG in the context of PIMS-TS has not been empirically verified since our report is limited regarding the small number of cases. Second, PIMS-TS in our two patients appeared in times of the emerging predominance of SARS-CoV-2 B1.1.7. The appearance of B1.1.7 in Germany correlated with rising numbers of PIMS-TS (16). Currently, in winter 2022/2023, omicron subvariants prevail and the incidence of PIMS-TS has been decreasing in Germany since the beginning of 2022 (16). Yet, it remains unpredictable if PIMS-TS will come up again due to new virus variants of SARS-CoV-2.

## Data Availability

The original contributions presented in the study are included in the article/Supplementary Material, further inquiries can be directed to the corresponding author.
